# PICK1 links AMPA receptor stimulation to Cdc42

**DOI:** 10.1016/j.neulet.2014.11.046

**Published:** 2015-01-12

**Authors:** Daniel L. Rocca, Jonathan G. Hanley

**Affiliations:** School of Biochemistry, Medical Sciences Building,University of Bristol, University Walk, Bristol BS8 1TD, UK

**Keywords:** Rho-family GTPase, Actin cytoskeleton, PDZ domain, BAR domain, Glutamate receptor

## Abstract

Rho-family GTPases control numerous cell biological processes via effects on actin dynamics, such as cell migration, cell adhesion, morphogenesis and vesicle traffic. In neurons, they are involved in dendritic spine morphogenesis and other aspects of neuronal morphology via regulation of the actin cytoskeleton. The Rho-family member Cdc42 regulates dendritic spine morphology via its effector N-WASP, which activates the actin-nucleating Arp2/3 complex. Excitatory synaptic transmission is known to regulate actin dynamics in dendritic spines to bring about changes in spine morphology or motility, however, the details of the signalling pathways that transduce glutamate receptor activation to Rho GTPase function are unclear. PICK1 is a PDZ and BAR domain protein that interacts with the Arp2/3 complex and the GTPase Arf1 to regulate actin polymerisation in dendritic spines. PICK1 also binds AMPA receptor subunits GluA2/3 and is involved in GluA2-dependent AMPAR trafficking. Here, we show that PICK1 binds Rac1 and Cdc42, via distinct but overlapping binding sites. Furthermore, AMPAR stimulation deactivates Cdc42 and alters its detergent solubility in neurons via a PICK1-dependent process. This work suggests a novel role for PICK1 in transducing AMPAR stimulation to Cdc42 function in neurons.

## Highlights

• PICK1 binds Rac1 and Cdc42.
• AMPA receptors can interact with Cdc42 via PICK1.
• AMPA stimulation increases Cdc42 detergent solubility in a PICK1-dependent manner.

## 1 Introduction

Rho-family GTPases are proteins of fundamental importance in integrating intracellular signalling pathways. They are molecular switches, cycling between an active GTP-bound state and an inactive GDP-bound state, and once activated bind to a wide range of effectors to initiate a diverse array of signalling pathways that control numerous cell biological processes via effects on actin dynamics, such as cell migration, cell adhesion, morphogenesis and vesicle traffic [10,23]. Rho GTPases are activated mainly through cell-surface receptors via guanine nucleotide exchange factors (GEFs), which promote GTP loading. Conversely, GTPase-activating proteins (GAPs) deactivate Rho GTPases by enhancing their enzymatic activity and returning the protein to a GDP-bound state [3,25,31].

In neurons, Rho-family GTPases such as RhoA, Rac1 and Cdc42 are involved in dendritic spine morphogenesis and other aspects of neuronal morphology via regulation of the actin cytoskeleton [21,31,32]. For example, Cdc42 regulates dendritic spine morphology via its effector N-WASP, which promotes actin polymerisation via activation of the Arp2/3 complex [14,33]. Glutamatergic synaptic transmission is known to regulate actin dynamics in dendritic spines [8,19], however, the details of the signalling pathways and molecular mechanisms that transduce glutamate receptor activation to Rho GTPase function are unclear.

Postsynaptic AMPA-type glutamate receptors (AMPARs) mediate most fast excitatory synaptic transmission and are crucial for many aspects of brain function, including learning, memory and cognition [6,17]. AMPARs undergo constitutive and activity-dependent trafficking to, and removal from, synapses. Changes in synaptic AMPAR number, subunit composition and/or channel properties result in long-term potentiation (LTP) or long-term depression (LTD) of synaptic efficacy [1,13].

PICK1 is a PDZ and BAR domain protein that interacts with the actin-nucleating Arp2/3 complex and inhibits actin polymerisation [12,24]. PICK1 binds AMPAR subunits GluA2/3 and is required for GluA2-dependent AMPAR trafficking in hippocampal neurons during synaptic plasticity, and also following pathological insults such as oxygen/glucose deprivation [7,30]. PICK1 also restricts dendritic spine size via Arp2/3 inhibition, and is involved in spine shrinkage during LTD [20]. ABP/GRIP is a family of multi-PDZ domain scaffold proteins that also interact with AMPAR subunits GluA2/3, and are involved in AMPAR trafficking [5,26].

Here, we show that PICK1 binds Rac1 and Cdc42, via distinct but overlapping binding sites. Furthermore, AMPAR stimulation deactivates Cdc42 and alters its detergent solubility in neurons via a PICK1-dependent process.

## 2 Materials and methods

### 2.1 Plasmids

Both pRK5–myc-Rac1 and pRK5–flag-Cdc42 were kind gifts from Prof. Kate Nobes. GST-Rhotekin, GST–PAK–CRIB and pcDNA3.1 myc-RhoA were kind gifts from Prof. Harry Mellor. All constructs were expressed in COS7 cell lines following transfection using Lipofectamine 2000 as per manufacturers instructions (Invitrogen). Sindbis virus constructs encoding the short peptides pep2-SVKI, pep2-SVKE and pep2-EVKI and EGFP after an IRES cassette were a kind gift from Prof. Jeremy Henley [29]. GST-R2C and his_6_-PICK1 were expressed in *Escherichia coli* BL21 and have been described previously (Hanley et al., 2002).

### 2.2 Antibodies

The antibodies used were as follows: anti-myc (9E10, Santa Cruz); anti-flag (M2, Sigma); anti-Cdc42 (clone44/CDC42, BD biosciences); anti-Rac1 (610,650, BD biosciences); anti-PICK1 (75-040, Neuromab) and anti-β-tubulin (clone TUB2.1, Sigma).

### 2.3 Primary neuronal culture and Sindbis virus transduction

Primary cortical neuronal cultures were prepared from E18 wistar rats as previously described (Hanley and Henley, 2005). Sindbis viruses were prepared as directed by the manufacturers instructions (Invitrogen). Infections were carried out around 20 h before cell lysis and were carried out as described before (Hanley et al., 2002)

### 2.4 Co-immunoprecipitations

Co-immunoprecipitations were carried out from dissociated cortical cultures as previously described [15]. Briefly cortical neurons were lysed in lysis buffer (0.5% TX-100, 150 mM NaCl, 20 mM Tris pH 7.5 plus protease inhibitors), the Triton X-100 content was then diluted to 0.25% using 150 mM NaCl and 20 mM Tris, pH 7.5, before immunoprecipitation with 2 μg control IgG, anti-PICK1 or anti-GluA2 antibodies. Bound proteins were detected by western blotting.

### 2.5 GST pulldowns

These were carried out as previously described [24]. GST–PICK1, GST–PAK–CRIB or GST–Rhotekin were incubated with lysates prepared from COS7 cells expressing epitope-tagged GTPases or with purified his_6_-tagged proteins. Bound proteins were detected by western blotting.

### 2.6 Cdc42 activation assays

Cortical neurons were stimulated with 100 μm AMPA for 5 min before lysis in 0.5% TX-100, 150 mM NaCl, 10 mM HEPES pH 7.4 and protease/phosphatase inhibitors. Subsequently, GTP-bound Cdc42 was isolated from lysates via a one-step batch purification using GST–PAK–CRIB pulldowns followed by western blotting. Total Cdc42 was also determined using a fraction of the neuronal extracts before pulldowns.

### 2.7 Western blot analysis and quantification

Western blots from five independent experiments were scanned and analysed by densitometry using ImageJ. error bars represent s.e.m., and two-tailed *t*-tests were carried out to determine significant differences between two conditions.

## 3 Results and discussion

**Fig.1:**
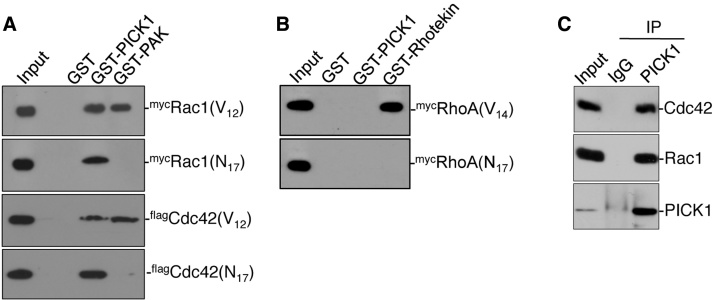
PICK1 interacts with Cdc42 and Rac1 but not RhoA. (A) PICK1 binds Rac1 and Cdc42. GST-pulldowns were carried out from lysates prepared from COS7 cells expressing myc-tagged Rac1(V_12_), Rac1(N_17_), Cdc42(V_12_) or Cdc42(N_17_) using GST–PICK1, GST–PAK–CRIB or GST alone. Bound proteins were detected by western blotting using anti-myc. (B) PICK1 does not bind RhoA. GST-pulldowns were carried out from lysates prepared from COS7 cells expressing myc-tagged RhoA(V_14_) or RhoA(N_17_) using GST–PICK1, GST-Rhotekin or GST alone. Bound proteins were detected by western blotting using anti-myc. (C) Cdc42 and Rac1 interact with PICK1 in neurons. Lysates prepared from cultured cortical neurons were immunoprecipitated with anti-PICK1 antibody or non-immune IgG as a control, and bound proteins were detected by western blotting using specific antibodies as shown.

To investigate the interaction of PICK1 with Rho-family GTPases, we carried out pulldown assays using GST–PICK1 and lysates prepared from COS cells expressing epitope-tagged Cdc42, Rac1 or RhoA. Since an important functional feature of Rho-family GTPases is that they bind downstream effector proteins preferentially in their active, GTP-bound state [4], we tested constitutively active (CA, V_12_) and dominant negative (DN, N_17_) mutant GTPases. p21 activated kinase (PAK) is a known effector for Cdc42 and Rac and binds CA but not DN mutants of both GTPases ([35] and Fig. 1A). GST–PICK1 binds CA and DN mutants equally well for both Rac1 and Cdc42 (Fig. 1A), suggesting that PICK1 is not a Rac1/Cdc42 effector, but perhaps plays a scaffolding role to localise the GTPases to specific subcellular locations. We carried out equivalent experiments for RhoA, using the known effector protein Rhotekin as a positive control [22]. GST–PICK1 does not interact with either RhoA mutant, demonstrating specificity for the interaction with Rac1 and Cdc42 (Fig. 1B). To confirm that Cdc42 and Rac1 interact with PICK1 in neurons, we carried out co-immunoprecipitations (co-IPs) from lysates prepared from cultured cortical neurons using anti-PICK1 antibodies. Both Rac1 and Cdc42 show a robust interaction with PICK1 (Fig. 1C), demonstrating that both GTPases interact with PICK1 in neurons.

**Fig. 2:**
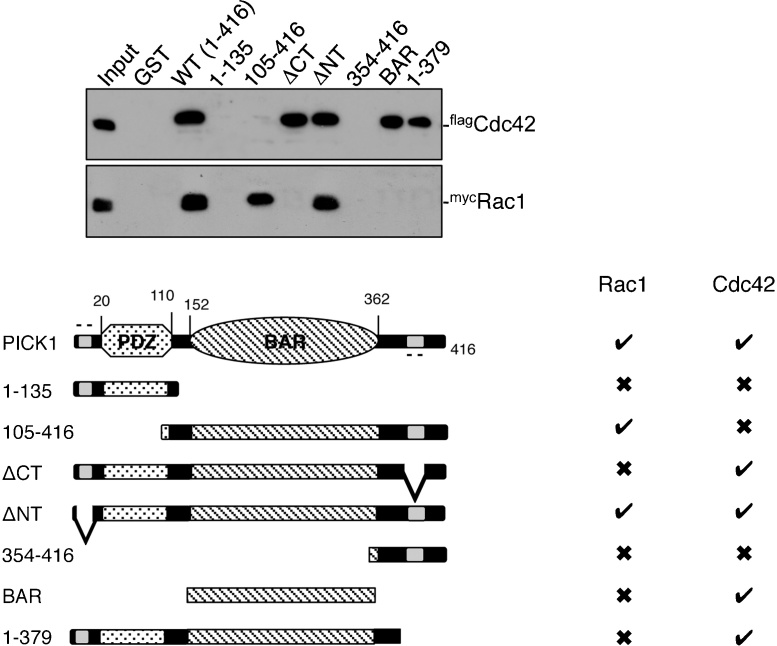
Cdc42 and Rac1 have distinct but overlapping binding sites on PICK1. Upper panel: GST pulldowns were carried out using purified his_6_^flag^Cdc42 or his_6_^myc^Rac1 and truncation mutants of PICK1 as GST fusions as depicted. Bound proteins were detected by western blotting using anti-myc or anti-flag. Lower panel: diagram showing truncation mutants of PICK1 used, and a summary of the results. A tick indicates a positive interaction, whereas a cross indicates no binding.

To further compare, the PICK1–Cdc42 interaction with that of PICK1–Rac1, we analysed the binding of purified his_6_-tagged ^flag^Cdc42 and ^myc^Rac1 to a range of PICK1 truncations. Wild-type GST–PICK1 binds both GTPases, demonstrating that the interactions are direct, with no requirement for intermediary protein components. Interestingly, the two GTPases show distinct patterns of binding to the PICK1 mutants, indicating that Cdc42 and Rac1 have overlapping, but not identical binding sites on PICK1 (Fig. 2). Both GTPases require the presence of the BAR domain, indeed Cdc42 binds the isolated BAR domain and binding is unaffected by deletion of either acidic region (ΔCT, ΔNT) or deletion of an extreme C-terminal region (1-379) of the full-length protein. However, Cdc42 binding is abolished in the absence of the PDZ domain when the C-terminal region is present (105-416), suggesting an intramolecular inhibition of the interaction. It has previously been suggested that PICK1 forms an intramolecular interaction between the PDZ and BAR domains [18,24], and also that the C-terminal tail interacts with the BAR domain [16]. In contrast, Rac1 does not bind the isolated BAR domain, but requires the presence of both BAR and C-terminal regions for the interaction (Fig. 2).

These results demonstrate that PICK1 directly interacts with Cdc42 and Rac1 via the BAR domain, with additional sequence determinants that suggest overlapping but distinct binding sites on PICK1. Rac1 has previously been shown to interact with Arfaptin BAR domain, which shows some homology to that of PICK1 [27,28]. The structure of the Rac–Arfaptin complex has been defined, and indicates that the GTPase sits on the concave face of the crescent-shaped BAR domain [28]. If a similar conformation exists for PICK1, this would suggest that GTPase binding and curved membrane binding to the BAR domain would be mutually exclusive. PICK1-bound GTPase would therefore, likely be cytosolic unless associated with a transmembrane protein.

**Fig. 3:**
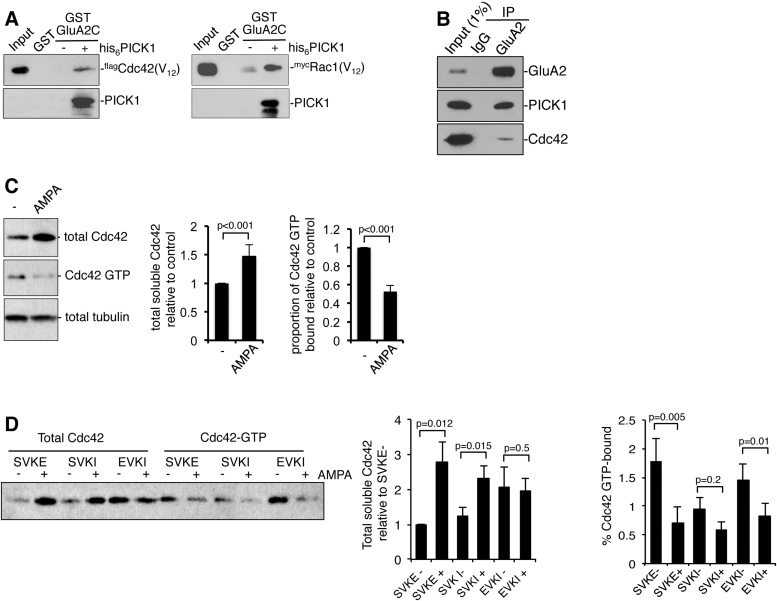
PICK1 links AMPAR stimulation to Cdc42 deactivation. (A) Both Cdc42 and Rac1 form a triple complex with PICK1 and GluA2 in vitro. GST-pulldowns were carried out from lysates prepared from COS7 cells expressing flag-tagged Cdc42(V_12_) or myc-tagged Rac1(V_12_) using GST-GluA2 C-terminus (GluA2C) in the absence or presence of purified his_6_PICK1, or GST alone. Bound proteins were detected by western blotting using anti-PICK1, anti-flag or anti-myc. (B) Cdc42 forms a triple complex with PICK1 and GluA2 in neurons. Lysates were prepared from dissociated cortical neurons, and immunoprecipitations carried out using anti-GluA2 or non-immune mouse IgG as control. Bound proteins were detected by western blotting using anti-GluA2, anti-PICK1 and anti-Cdc42. (C) AMPAR stimulation increases the detergent solubility of Cdc42 and reduces the proportion of GTP-bound Cdc42. Dissociated cortical neurons were treated with 100 μM AMPA or vehicle for 5 min. Lysates were prepared and GTP-bound Cdc42 was isolated by GST pulldown using GST–PAK. GST–PAK bound Cdc42–GTP and unbound Cdc42 in the lysate were detected by western blotting using anti-Cdc42. Tubulin serves as a loading control. Representative western blots are shown, and graphs show pooled data for total detergent-soluble Cdc42 (left graph) and for the proportion of Cdc42 that is GTP-bound (right graph). *n* = 5. (D) PICK1 PDZ domain interactions are involved in AMPAR-induced changes in detergent solubility of Cdc42. Dissociated cortical neurons were transduced with Sindbis virus expressing pep2-SVKE–IRES-EGFP, pep2-SVKI–IRES-EGFP or pep2-EVKI–IRES-EGFP. Cultures were treated with AMPA(+) or vehicle(−), and processed for biochemistry as in (B). A representative western blot is shown, and graphs show pooled data for total detergent-soluble Cdc42 (left graph) and for the proportion of Cdc42 that is GTP-bound (right graph). *n* = 5.

Since PICK1 is a well-established AMPAR accessory protein [12], we explored an association between AMPAR stimulation and Cdc42. Initially, we investigated whether PICK1 can form a triple complex with Cdc42 or Rac1 and GluA2 C-terminus. GST–GluA2 C-terminus (GluA2C) does not bind Cdc42 or Rac1 in the absence of PICK1, but when his_6_-PICK1 is added, a robust interaction with both GTPases is observed (Fig. 3A). Furthermore, both Cdc42 and PICK1 are present in GluA2 immunoprecipitations from neuronal lysate, strongly suggesting the presence of a GluA2–PICK1–Cdc42 tripartite complex in vivo (Fig. 3B). These experiments demonstrate that Cdc42 can associate with AMPARs via PICK1, and suggest that either Cdc42 regulates AMPAR trafficking, or AMPARs regulate Cdc42 function via PICK1. To test the latter hypothesis, we used GST-PAK pulldown assays to determine the effect of AMPAR stimulation on Cdc42 activation in cultured neurons. Bath application of AMPA for 5 min causes a significant reduction in GTP-bound Cdc42 (Fig. 3C). In addition, we noted an increase in the detergent solubility of Cdc42 after AMPAR stimulation (Fig. 3C), suggesting that AMPAR stimulation displaces Cdc42 from specific membrane compartments or protein complexes. Since cell lysis and western analysis were carried out after just 5 min of drug treatment, it is highly unlikely that this difference in Cdc42 immunoreactivity could be explained by an increase in protein translation or a reduction in protein degradation.

To investigate whether PICK1 could mediate these effects of AMPA on Cdc42, we used Sindbis virus to express peptides mimicking the C-terminus of GluA2 that block AMPAR–PDZ domain interactions, and hence, disrupt the link between Cdc42 and AMPARs. Pep2-SVKI represents the wild-type sequence and disrupts PDZ interactions with PICK1 and ABP/GRIP, whereas pep2-EVKI is selective for PICK1. Pep2-SVKE does not bind PDZ domains, and hence, serves as a negative control [29]. Interestingly, the presence of pep2-EVKI increases the detergent-solubility of Cdc42 under basal conditions, which occludes the effect of subsequent AMPAR stimulation. However, pep2-EVKI has no effect on AMPA-induced Cdc42 deactivation, which is similar to pep2-SVKE expressing neurons (Fig. 3D). In contrast, pep2-SVKI has no effect on detergent solubility, but causes a decrease in GTP-bound Cdc42, which occludes the effect of AMPA application.

These results suggest that PICK1 is involved in the change in subcellular localisation of Cdc42 that occurs as a result of AMPAR stimulation. Since disrupting GluA2–PICK1 binding causes the same change in detergent solubility as stimulating AMPARs, these results are consistent with a model whereby a Cdc42–PICK1–AMPAR complex is associated with TX-100 resistant membrane compartments or protein complexes under basal conditions, and dissociates upon AMPAR stimulation. Following dissociation of PICK1 from membrane-bound AMPARs, Cdc42 would have a more cytosolic distribution, and would consequently be more detergent-soluble. Our results also suggest a role for ABP/GRIP in AMPAR-dependent changes in Cdc42 activation. Since pep2-SVKI but not pep2-EVKI occlude the effect of AMPAR stimulation, dissociation of ABP/GRIP from AMPARs may be involved in AMPA-induced Cdc42 deactivation. A potential explanation for this result is that PICK1 binds ABP/GRIP [18] and can therefore, associate with AMPARs independently of the PICK1 PDZ domain, but via the ABP/GRIP PDZ domain. It is therefore, possible that a GluA2–GRIP–PICK1–Cdc42 complex is involved in regulating Cdc42 activity. Although there are no reports of Cdc42, GAPs or GEFs that associate with ABP/GRIP, GRASP-1 is a Ras GEF that binds directly to GRIP1, indicating that such a mechanism is feasible [34]. Although the mechanistic details are likely to be different, this model may have some features in common with the functional effect of RhoGDIs on Rho-family GTPases. RhoGDIs associate with cytosolic GTPases, blocking their association with membranes, and maintaining the GTPase in an inactive, GDP-bound state [9].

PICK1 inhibits actin polymerisation by direct binding to the Arp2/3 complex. Activated (GTP-bound) Arf1 attenuates Arp2/3–PICK1 binding, hence, PICK1 is a downstream effector of the small GTPase in this pathway. In the current study, we show that Cdc42 binds PICK1 in a GTP-independent manner and that PICK1 is upstream of Cdc42. Hence, PICK1 can function in multiple ways to regulate actin dynamics in neurons, and is one of a growing number of BAR domain containing proteins that have critical roles in controlling the actin cytoskeleton in multiple cell types [2]. It will be of great interest to determine the precise function of Rac1/Cdc42 binding to PICK1 in the control of actin-dependent processes in neuronal function.

In conclusion, our results suggest that AMPAR activation regulates Cdc42 subcellular localisation and function via PICK1. This could provide a mechanism for the regulation of local actin polymerisation in dendritic spines to regulate spine dynamics or morphology. Consistent with this hypothesis, it has been shown that AMPAR stimulation blocks spine motility and causes alterations in actin polymerisation leading to morphological changes in spines that are believed to correspond to spine stabilization and maturation [8,11].
